# Therapeutic Efficacy of E-64-d, a Selective Calpain Inhibitor, in Experimental Acute Spinal Cord Injury

**DOI:** 10.1155/2015/134242

**Published:** 2015-07-09

**Authors:** Zifeng Zhang, Zheng Huang, Hao Dai, Licheng Wei, Songtao Sun, Feng Gao

**Affiliations:** ^1^Department of Spine Surgery and Traumatology, Guanghua Integrative Medicine Hospital, Shanghai 200052, China; ^2^Institute of Arthritis Research, Shanghai Academy of Chinese Medical Sciences, Shanghai 200052, China

## Abstract

This study aims to investigate the therapeutic effect of calpain inhibitor E-64-d on SCI and to find a new approach to treat SCI. When an SCI rat model was established, it was immediately administered with E-64-d. RT-PCR and Western blotting were used to determine the protein and mRNA levels of calpain 1 and 68-kD NFP. TUNEL staining and NeuN labeling were performed to analyze neuronal apoptosis in the lesion. Immunohistochemistry assay was carried out to observe the expressions of calpain 1 and GFAP. Cyclooxygenase-2 activity was measured to show the immune response status. Locomotor function was evaluated by inclined plane test and Basso, Beattie, and Bresnahan locomotor rating scale. The results showed that calpain 1 was activated after SCI occurred. Treatment with E-64-d decreased expressions of calpain 1 and GFAP, alleviated neuronal apoptosis, inhibited cyclooxygenase-2 activity, and resulted in the promoted locomotor function. Furthermore, combination of E-64-d and MP had better efficacy than did E-64-d or MP alone. E-64-d is expected to be applied to treat SCI, and its alliance with MP may provide a valid strategy for SCI therapy.

## 1. Introduction

Spinal cord injury (SCI) is the most serious complication of spinal injury, which often leads to the severe dysfunction of limbs and trunk below the damaged section, and it is a common cause of permanent disability and death in both children and adults [1, 2]. Acute spinal cord injury is due to a traumatic injury that can result in a bruise (contusion), a partial tear, or a complete tear (transection) in the spinal cord. The secondary injury process initiated by primary injury to the spinal cord includes the activation of various cysteine proteases for degradation of cytoskeletal protein and other crucial proteins for delayed death of neurons and glial cells in the lesion and adjacent areas [3].

Calpains belong to the family of calcium-dependent, nonlysosomal cysteine proteases expressed ubiquitously in mammals. They are implicated in cellular key cytoskeleton degradation and neurodegeneration at the site of SCI and its penumbra [4]. Inhibiting calpain expression with the cell-permeable, irreversible cysteine protease inhibitor E-64-d can prevent apoptosis and restore transcription of proteolipid protein and myelin basic protein genes, which indicates the therapeutic efficacy of E-64-d for treatment of SCI [5, 6]. Methylprednisolone (MP), a synthetic glucocorticoid drug, is typically used for its anti-inflammatory effects, and it is also prescribed for SCI because it improves sensory and locomotor recovery if given within 8 h of the injury [7]. However, the controversy still exists in the use of MP in the treatment of SCI due to the lack of controlled data about the long-term effects of treatment [3, 8–10].

Previously, we focused our study on the effects of E-64-d and MP on the spinal cord ischemia-reperfusion injury [11–13] and found both E-64-d and MP could suppress the expression of calpain 1 and protect the spinal cord tissue from the secondary injury to different extents [13]. Although some studies have already covered the effect of E-64-d on SCI in some respects, we still wonder more sufficient knowledge about the mechanism and what the outcome will be if it is in alliance with MP.

## 2. Materials and Methods

### 2.1. Experimental Animals and Grouping

Male Sprague Dawley (SD) rats weighing 220 ± 20 g were purchased from Shanghai Laboratory Animal Center, Chinese Academy of Science, China. They were randomly divided into 5 groups: (A) sham group, underwent laminectomy only; (B) SCI group, spinal cord injured without any treatment; (C) E-64-d treatment group, spinal cord injured and immediately intravenously injected with E-64-d (1 mg/kg, using 1.5% DMSO as vehicle); (D) methylprednisolone (MP) treatment group, spinal cord injured and immediately intravenously injected with MP (165 mg/kg); (E) E-64-d + MP treatment group, spinal cord injured and immediately treated with both E-64-d (1 mg/kg) and MP (165 mg/kg). Every treatment group was treated with the corresponding drug for 7 consecutive days, and sham and SCI groups, which were, respectively, intended as blank and negative controls, were treated with the same amount of 1.5% DMSO. In each group, 3 rats were left and continued receiving the aforesaid treatment for another 7 days, which were used for locomotor function tests.

### 2.2. Rat SCI Model

Spinal cord of each rat in group (B), (C), (D), and (E) was injured using a modified Allen's weight-drop method as previously described [14–16]. In brief, laminectomy was performed at T10 in anesthetized rats under a dissecting microscope, and then the rats were fixed on a stereotactic device. After the spinal column was immobilized, 0.3 cm diameter impounder was gently placed on the spinal cord. To make the injury, a constant weight (5 g) from a height of 8 cm was dropped onto the impounder. After surgery, penicillin (4 × 10^4^ U once; twice per day) was intramuscularly injected for 3 consecutive days to prevent postoperative infection. In the follow-up experiments, tramadol hydrochloride (15 mg/(kg · d)) was added to the drinking water provided for the rats to relieve their pain. In addition, artificial auxiliary urination was given when rats' temporary urinary retention occurred in the early postoperative phase until the rats started automatic micturition.

### 2.3. Total RNA Extraction and RT-PCR

Total RNA was extracted from the spinal cord tissue at T10 using Trizol Reagent (Qiagen, Valencia, OH), and then RT-PCR analysis was performed. In brief, cDNA of calpain 1 was synthesized from 2 *μ*g total RNA using High Capacity RNA to cDNA Kit (Applied Biosystems, CA, USA). TaqMan Fast Advanced Master Mix (Applied Biosystems, CA, USA) was used for PCR. The primers used for quantitation of the cDNAs were as follows.

*Calpain 1 [4]*

 5′-CACTTGAAGCGTGACTTCTTCCTGGCCAATGC-3′ (forward primer),
 5′-GCACTCATGCTGCCCGACTTGTCCAGGTCAAACTT-3′ (reverse primer).

*GAPDH*

 5′-TCTTGTGCAGTGCCAGCCTC-3′ (forward primer),
 5′-CACGCCACAGCTTTCCAGAG-3′ (reverse primer).

The amplification conditions were as follows: 94°C for 3 min, followed by 35 cycles at 94°C for 20 s, 58°C for 20 s, and 72°C for 30 s. The mRNA expression of calpain 1 was calculated using the comparative threshold cycle (CT) and normalized to that of GAPDH (DCT). The above procedures were repeated thrice and all the samples were performed in triplicate.

### 2.4. Mitochondria and Cytosol Isolation

A tissue mitochondria isolation kit (Beyotime, China) was used for mitochondria and cytosol isolation according to its instructions. Briefly, after the fresh spinal cord tissue at T10 was well ground in an ice-bath, the homogenate was centrifuged at 1000 g and 4°C for 5 min. The supernatant (cytosol) and precipitate (mitochondria) were, respectively, collected. The related protein AIF in cytosol and mitochondria was subsequently analyzed using Western blotting.

### 2.5. Western Blotting

Western blot analysis was performed referring to the previous report [17]. In brief, the spinal cord tissue at T10 section was collected and homogenized in protein extraction reagent. After the homogenate was centrifuged at 1,2000 rpm for 10 min at 4°C, the protein concentration was quantified using a BCA Protein Assay Kit (Pierce Chemical Company, Rockford, IL, USA) according to the manufacturer's protocol. Protein samples (80 *μ*g for each) were loaded onto SDS-polyacrylamide gel after denaturation in boiling water for 5 min. The samples were transferred to the nitrocellulose membrane after electrophoresis. The blots were firstly incubated with the primary IgG antibodies (1 : 1000 dilutions) against calpain 1, 68-kD NFP, AIF, MAP1B, VADC, and *β*-actin, and then incubated with the goat-anti-rabbit secondary antibody IgG antibody. The bands were illuminated by an ECL system (Santa Cruz Biotechnology, Santa Cruz, CA, USA) and autoradiography assay. These procedures were repeated three times. The ECL autoradiograms were imaged on a UMAX Powerlook scanner and band intensities were determined densitometrically using Adobe PhotoShop software (version CS5, Adobe Systems, San Jose, CA, USA). The ratios of the density of the above proteins to *β*-actin or VADC were calculated for each sample.

### 2.6. Terminal Deoxynucleotidyl Transferase-Mediated Deoxyuridine Triphosphate-Biotin Nick End Labeling (TUNEL) and Neuronal Labeling

Referring to the literature [18], TUNEL and neuronal labeling were successively performed. In brief, serial 6 *μ*m paraffin sections were dried at 62°C in an air oven for 2 h, after which the sections were dewaxed with xylene, hydrated with ethyl alcohol, and washed thrice with PBS for 3 min (each time). The samples were incubated with proteinase K at room temperature for 30 min and then washed again with PBS as ditto. In order to inhibit EGPO (endogenous peroxidase), H_2_O_2_ (3%) was added to the samples and incubation at room temperature was performed for 20 min. After washing with PBS 3 times, the sections were incubated in equilibration buffer at room temperature for 20 min. Bibulous paper was used to absorb most of the equilibration buffer, and TdT (terminal deoxynucleotidyl transferase) incubation buffer was added for the following incubation at 37°C for 1 h. The sections were washed with PBS, following which serum was added and incubation at 37°C lasted for 30 min. Subsequently, incubation at 4°C overnight after the addition of primary antibody (anti-NeuN, 1 : 300; Abcam Plc, Cambridge, UK) and incubation at 37°C for 1 h after the addition of secondary antibody (donkey anti-mouse IgG H&L (DyLight 650), 1 : 200; Abcam, UK) were successively performed. Besides, all cell nuclei were stained with DAPI (4′,6-diamidino-2-phenylindole; Biohao Biotec Co., Ltd., Shanghai, China) for 20 min. After PBS washing, the reagent (Beyotime Biological Technology Ltd., Shanghai, China) that prevents fluorescence quenching was added. The sections were mounted and reserved at −20°C.

Images of the fluorescent immunohistochemistry were photographed at 200x magnification under a fluorescence microscope (Olympus BX51, Olympus, Melville, NY, USA). In each photo, the stained cells were manually counted using AxioVision 4.2 software (Carl Zeiss, Thornwood, NY, USA). The cells were counted by 3 researchers who had no knowledge of the treatment status of each rat.

### 2.7. Measurement of Caspase-3 Activity

The enzyme activity of caspase-3 in the fresh tissue was measured using a caspase-3 colorimetric assay kit (Beyotime, China). According to the protocols, every 3~10 mg tissue was lysed by 100 *μ*L lysis buffer and ground in an ice-bath for 5 min. After centrifugation at 20,000 g and 4°C for 10 min, the supernatant was transferred to precooled centrifuge tube for the immediate activity detection of caspase-3. The enzyme reaction was performed in a 96-well flat-bottomed microplate and quantified using a microplate reader.

### 2.8. Immunohistochemical Analysis of Calpain 1 and GFAP

The paraffin sections were dried in an air oven (58°C) for 2~4 h. The tissues were dewaxed with xylene and hydrated with alcohol. After the sections were washed with PBS, antigen retrieval solution (0.01 mol/L sodium citrate, pH = 6.0) was added; then they were boiled at a constant temperature of 95°C for 15 min. When the sections were naturally cooled down, PBS was used again to wash them. The tissues were incubated with H_2_O_2_ (3%) at room temperature for 10 min for the inhibition of EGPO. They were washed with PBS, and 10% fetal calf serum was added. After 30 min of the incubation, the sections were washed with PBS thrice. Primary antibody (Abcam Plc, Cambridge, UK), namely, anti-calpain 1 (1 : 400) or anti-GFAP (1 : 50), was added for the following incubation at 4°C overnight. After secondary antibodies (DAKO, Copenhagen, Denmark) were added, incubation at 37°C was kept for 1.5 h. Conventional DAB development (3~5 min) and hematoxylin counterstaining (30 s) were successively performed. After dehydration and drying, the sections were mounted with resin. Images were taken under 200x magnification using a Nikon Eclipse 50i microscope (H550S) (Nikon Inc., Melville, NY, USA).

### 2.9. Determination of Cyclooxygenase-2 (COX-2) Activity

Using a rat COX-2 ELISA kit (Shanghai Westang Bio-Tech Co., Ltd., Shanghai, China), double-antibody sandwich ABC-ELISA method was applied to detect the activity of rat's COX-2. According to the instructions, the ELISA plate was coated with rat COX-2 monoclonal antibody, and then COX-2 in the standards or samples was binding to the monoclonal antibody. After biotinylated anti-COX-2 was added, immune complex was formed on the plate. Then HRP (horse radish peroxidase) labeled streptavidin was combined with biotin. In the end, the substrate working solution was added to make it blue and the reaction was terminated by sulfuric acid. Absorption value was measured at 450 nm. COX-2 concentration was in direct proportion to the absorbance; therefore the concentration of COX-2 in the samples could be calculated based on the standard curve.

### 2.10. Locomotor Function Assessment

#### 2.10.1. Rivlin's Inclined Plane Test

According to the reports [19, 20], we made a simple device containing a moveable plate with an adjustable angle of 0–90°. At 3 and 7 days after injection with different drugs, rat locomotor function was tested using the modified Rivlin's method [20]. The rat's head was placed faced forward, and the longitudinal axis of its body was put perpendicular to the longitudinal axis of the oblique plate. The angle of inclination gradually increased until the rats could maintain a constant position for just 5 s. The angle was considered to be the critical value and then recorded. Each rat was tested at least three times.

#### 2.10.2. Basso, Beattie, and Bresnahan Locomotor Rating Scale

In accordance with the literature [21, 22], the rats' behavior was observed and BBB scores were recorded after 14 days of different treatments. The scores were analyzed by repeated measures ANOVA using Tukey's multiple comparison test.

### 2.11. Statistical Analysis

All data were expressed as mean ± SD and statistically analyzed using one-way ANOVA followed by Fisher's post hoc test, and differences were considered significant at the *p* < 0.05 level.

## 3. Results

### 3.1. Calpain 1 Was Activated after SCI

**Figure 1:**
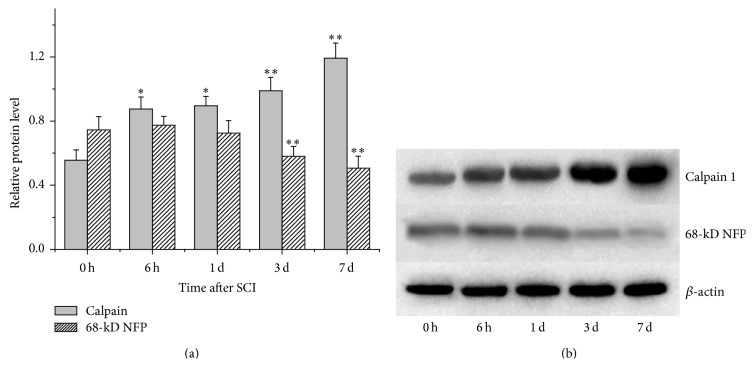
Protein expressions of calpain 1 and its substrate 68-kD NFP (nandrolone furylpropionate) in the lesion detected by Western blotting at 0 h, 6 h, 1 d, 3 d, and 7 d after SCI. (a) Semiquantitative results of Western blot analysis of calpain 1 and 68-kD NFP. (b) A representative result of Western blotting. ^*∗*^ *p* < 0.05, and ^*∗∗*^ *p* < 0.01, compared with the protein levels at 0 h.

After SCI was induced, the protein level of calpain 1 in the lesion significantly (*p* < 0.05) increased at 6 h and 1 d, and the differences became more significant (*p* < 0.01) during the following days (Figure 1(a)). As a substrate of calpain 1, 68-kD NFP was accordingly degraded (Figure 1(b)). Three and seven days after SCI induction, the degradation of 68-kD NFP was found significantly (*p* < 0.01) increased (Figure 1(a)).

### 3.2. The Expression of Calpain 1 Was Decreased by E-64-d

**Figure 2:**
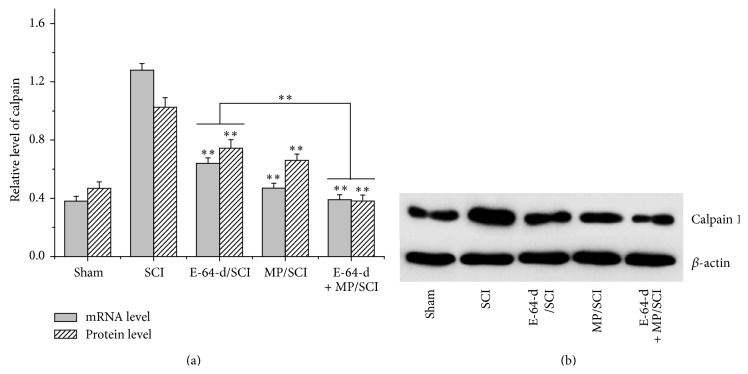
MRNA and protein levels of calpain 1 in the injured cord were measured with RT-PCR and Western blotting after SCI was differently treated for 7 d. (a) Quantitative mRNA level and semiquantitative protein level of calpain 1. (b) A representative image of Western blotting. ^*∗∗*^ *p* < 0.01, compared with SCI group; or E-64-d treatment versus E-64-d + MP treatment.

Calpain 1 mRNA and protein levels were both significantly (*p* < 0.01) reduced after the treatment with calpain inhibitor E-64-d or MP for 7 d (Figure 2(a)). In addition, the combined utilization of E-64-d and MP resulted in the significantly (*p* < 0.01) lower calpain 1 mRNA and protein levels compared to the treatment with only E-64-d.

### 3.3. Neuronal Apoptosis Was Reduced by E-64-d

**Figure 3:**
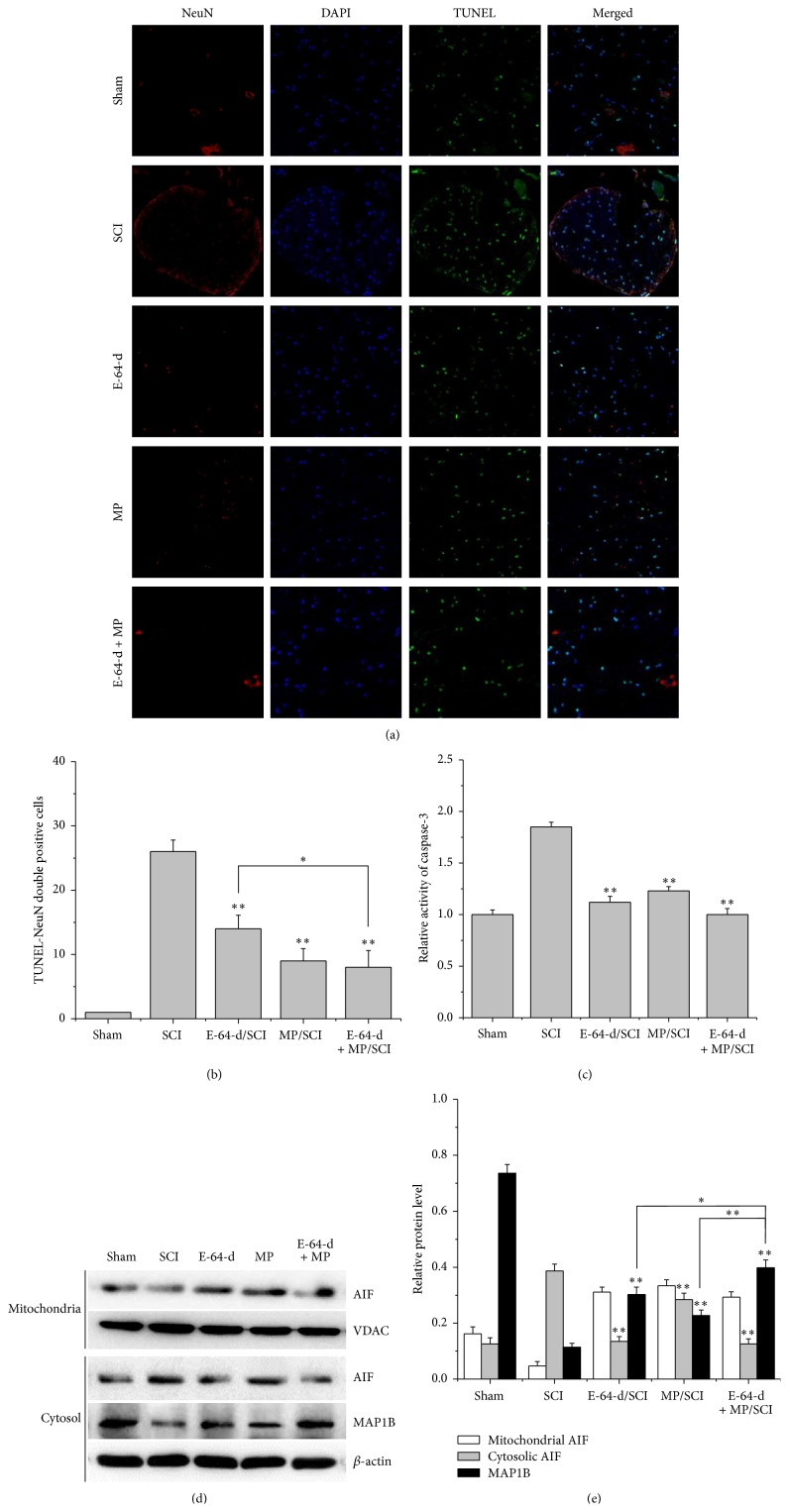
(a) Representative images of NeuN and TUNEL-stained sections of rats treated with different drugs for 3 d. (b) Quantification of NeuN and TUNEL double positive cells. (c) Activity of caspase-3 in injured spinal cord from each group. (d) Protein expressions of AIF and MAP1B analyzed by Western blotting. (e) The relative densities of AIF and MAP1B (*β*-actin and VADC served as internal references). ^*∗*^ *p* < 0.05, E-64-d treatment versus E-64-d + MP treatment; ^*∗∗*^ *p* < 0.01, compared with SCI group.

Three days after the SCI model rats had been subjected to E-64-d and MP, NeuN and TUNEL staining (Figure 3(a)) showed that the SCI rats treated with E-64-d or MP had significantly (*p* < 0.01) less NeuN-TUNEL-positive cells in the lesion than the rats in sham group had (Figure 3(b)). Compared with E-64-d treatment group, the group treated with both E-64-d and MP showed a significant (*p* < 0.05) decrease in neuronal apoptosis (Figure 3(b)). Consistently, caspase-3 was found activated in SCI group, compared with which E-64-d, MP, and E-64-d + MP treatment groups showed significantly (*p* < 0.01) lower activity of caspase-3 (Figure 3(c)).

In order to preliminarily explore the mechanism of E-64-d-induced neuronal apoptosis, we also measured AIF released from mitochondria and a microtubule-associated protein MAP1B, which is involved in an essential step in neurogenesis-microtubule assembly. The results indicated that cytosolic AIF released from mitochondria markedly increased after SCI, but the increase was effectively (*p* < 0.01) suppressed by different treatments, in which treatment with E-64-d alone or E-64-d associated with MP was found to be much better than treatment with only MP (Figures 3(d) and 3(e)). Additionally, MAP1B in SCI lesion degraded significantly (*p* < 0.01) 3 days after hit. But the treatments could remarkably (*p* < 0.01) slow down the degradation (Figures 3(d) and 3(e)). Notably, the combination of E-64-d and MP had a significantly (*p* < 0.05) better inhibitory effect on MAP1B degradation than E-64-d and MP used alone.

### 3.4. The Expressions of Calpain 1 and GFAP Were Reduced by E-64-d

**Figure 4:**
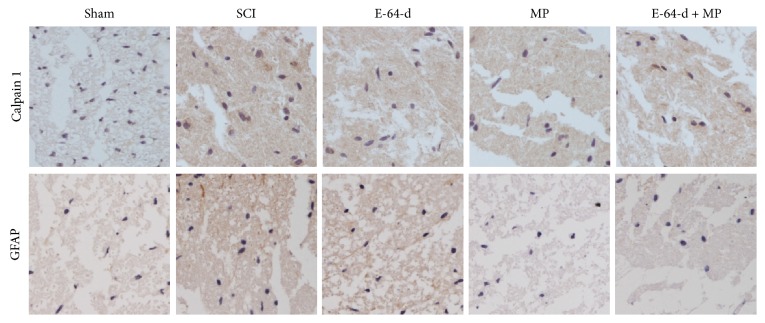
Calpain 1 and GFAP expression in the lesion of the spinal cord tissue detected by immunohistochemical staining after different treatments for 3 d.

As shown in Figure 4, the tissue from SCI model rats had evidently stronger staining, including both calpain 1- and GFAP-positive stains, than that from sham-operated rats. But, in the groups treated with E-64-d and MP, the expressions of calpain 1 and GFAP were remarkably less than those in SCI group. These results suggested that E-64-d could not only suppress calpain 1 expression but also somehow reduce the glial scar following SCI.

### 3.5. COX-2 Activity Was Inhibited by E-64-d

**Figure 5:**
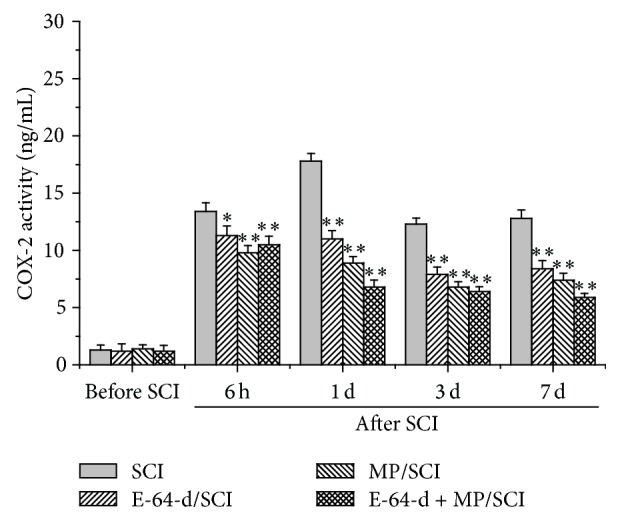
Enzyme activity of COX-2 in the injured spinal tissue before and after different treatments. ^*∗*^ *p* < 0.05, and ^*∗∗*^ *p* < 0.01, compared with SCI group.

As shown in Figure 5, after the spinal cord was injured, COX-2 activity sharply increased and peaked at 24 h. But the treatment with E-64-d or MP significantly reduced the enzyme activity, indicating that the inflammatory response in the spinal cord caused by injury could be suppressed by E-64-d or MP. What is more, the combination of E-64-d and MP had a better effect on the decrease in activity of COX-2 than did E-64-d or MP alone.

### 3.6. Locomotor Function Recovery of SCI Rats Was Promoted by E-64-d

**Figure 6:**
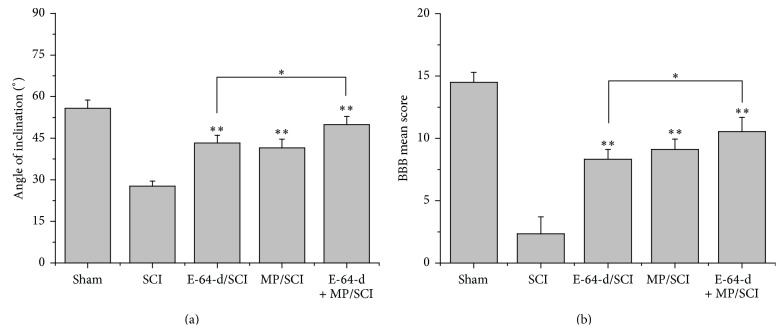
Locomotor function evaluated by inclined plane test (Rivlin's method, (a)) and BBB analysis (b) after different treatments for 2 weeks. Angle of inclination and the BBB mean score reflect locomotor function of rats' limbs. ^*∗*^ *p* < 0.05, E-64-d treatment versus E-64-d + MP treatment; ^*∗∗*^ *p* < 0.01, compared with SCI group.

To determine whether E-64-d and its combination with MP can improve recovery of locomotor function after T10 injury, locomotion was assessed using the inclined plane test (the modified Rivlin's method) and the BBB locomotor scale. SCI rats that were treated with E-64-d, MP, or both E-64-d and MP for 2 weeks exhibited significantly (*p* < 0.01) greater inclination and higher BBB locomotor function scores compared with SCI negative control animals (Figures 6(a) and 6(b)). Furthermore, we found that E-64-d + MP treatment had a significantly (*p* < 0.05) stronger efficacy than did E-64-d treatment.

## 4. Discussion

If the spinal cord is mechanically injured, a cascade of pathophysiological processes rapidly follows and results in secondary neuronal damage that will significantly exacerbate the original injury [23, 24]. Secondary spinal cord damage occurs within minutes and continues for days or even several weeks, leading to further neurological deterioration [25]. Calpain activation, cell apoptosis, inflammatory response, and excessive cytokine release at the site of the initial lesion are mainly responsible for the secondary spinal cord pathology [23, 26, 27].

Calpains are a class of enzymes containing an active-site cysteine residue that is important in protein degradation, which play the most prominent roles in causing both necrotic and apoptotic death in SCI lesion and penumbra [3, 28]. As mentioned above, cytoskeletal degradation and neurodegeneration feature prominently in the commitment of an injured neuron to a necrotic or apoptotic death [4, 29]. Additionally, they may induce apoptotic death by activating some apoptosis proteins like caspase-3 via an apoptosis pathway [28]. Therefore, prompt inhibition of calpains, if undertaken early enough after injury, could significantly spare lots of neurons. E-64-d is an inhibitor of cysteine proteases, including calpains [6], and it prevents calpain-mediated neuron apoptosis in the lesion and penumbra following spinal cord injury [4, 17, 30, 31]. In this study, not only was calpain 1 found to be gradually overexpressed after the spinal cord was injured, but also its activity was raised over time (Figure 1), which was proved by the degradation of the cytoskeletal protein 68-kD NFP. But both its mRNA and protein expression were inhibited by the immediate intravenous injection of E-64-d (Figures 2 and 4). The results of NeuN and TUNEL double staining showed a negative correlation between neuron apoptosis and E-64-d (Figure 3), indicating that inhibition of calpain 1 at least partly rescued neurons from apoptosis in the lesion and may be therefore beneficial to recovery of SCI. The underlying mechanism involved here is probably associated with the proapoptotic effect of activated calpain 1. It has been reported, in addition to inducing caspase-3 activation, that activated calpain-1 in neurons was also able to promote mitochondrial membrane permeability and cause AIF release from mitochondria [32]. In our present study, we found that E-64-d hindered the activation of caspase-3 and inhibited the release of AIF into cytosol, which is probably because of the significant inhibition of calpain 1 by E-64-d. As a substrate of both calpain 1 and caspase-3 [33, 34], MAP1B degradation was suppressed when the activation of calpain 1 and caspase-3 was directly or indirectly inhibited (Figures 3(c), 3(d), and 3(e)). It is known that MAP1B belongs to a type of neuron-specific proteins and it is the earliest expressed microtubule-associated protein during neurogenesis [35]. Normally, highly expressed MAP1B not only is necessary for microtubule assembly and the stability of microtubules but also means a lot for neural growth and regeneration [35, 36]. So, the degradation of MAP1B resulted from activated calpains and/or caspase-3 contributed significantly to the apoptosis of neurons as well, which suggested that inhibition of calpain 1 might be an effective method to attenuate SCI.

At the early stage of SCI, reactive astrocytes start to synthesize abundant GFAP and release various cytokines like NGF (nerve growth factor), bFGF (basic fibroblast growth factor), and IL (interleukin), which is in favor of recovery of neuron damage [37]. However, the glial scar resulted from excessive hyperplasia of astrocytes impedes axonal regeneration and growth [38]. GFAP (glial fibrillary acidic protein) can be used as a marker of astrocytes and its level reflects the neurologic damage degree [39]. Here we used immunohistochemical analysis to show that GFAP expression was remarkably reduced by E-64-d (Figure 4), suggesting that E-64-d may contribute to axonal regeneration and growth through attenuating glial scar formation.

The inflammatory response plays an important role in the development of secondary damage [40]. It is mediated by multiple molecular mechanisms, in which the formation of prostaglandins by COX-2 (prostaglandin H2 synthase) is one of the most prominent [41]. For this reason, COX-2 inhibitors have attained widespread use as anti-inflammatory agents, although potentially adverse side effects exist [41, 42]. COX-2 is an inducible immediate early gene that can be highly induced by inflammatory stimuli including cytokines evoked by spinal cord trauma [43, 44]. In addition, various studies have proved that the neuronal expression of COX-2 is directly toxic to neurons [44, 45]. In our present study, we found the sharp increase of COX-2 activity and expression after SCI, which was consistent with the study previously reported by other researchers [44, 46], and, therefore, the inflammatory response might be effectively relieved by E-64-d (Figure 5). According the related reports, E-64-d is able to alleviate inflammation through significantly decreasing IL-6 and IL-1*β* mRNA levels [47], and the calpain inhibitor can reduce the levels of cyclooxygenase-2 (COX-2) at the inflamed joints [48]. The molecular mechanism is probably closely associated with NF-*κ*B which can be activated by calpain through the degradation of I*κ*B [49], because COX-2 expression is induced by NF-*κ*B [47, 50].

SCI usually results in a change, either temporary or permanent, in normal locomotor, sensory, or autonomic function of the cord. After SCI, patients' locomotor function below the level of the injury can be badly damaged or even paralyzed [51]. The inclined plane test and 21-point BBB open field locomotor score are widely accepted for assessing locomotor recovery of the animals [52]. As shown in Figure 6, both the above tests indicated a better result of locomotor recovery in E-64-d treatment group than in SCI group. These results proved that E-64-d not only improved the microenvironment in the lesion but also, as a result, macroscopically promoted the locomotor function recovery of rats after SCI.

In addition, we combined the treatments of MP and E-64-d in the study because the use of MP in the treatment of SCI still remains highly controversial despite favorable results in randomized, controlled trials, and we are trying to explore a better strategy to successfully treat SCI for functional neuroprotection and preservation of locomotor function [3, 9]. As a whole, our present data showed that the combination of MP and E-64-d had a promising effect on SCI recovery, though some nonsignificant differences existed between MP treatment group and E-64-d + MP treatment group.

## 5. Conclusions

In conclusion, calpain activation is involved in rat SCI induced by weight drop, and immediate treatment with the selective calpain inhibitor E-64-d not only inhibited calpain 1 activation and COX-2 activity but also alleviated neuronal apoptosis partly through reducing the activation of caspase-3, AIF release, and degradation of MAP1B and decreased excessive hyperplasia of astrocytes. As a result, locomotor recovery after SCI was promoted. What is more, joint use of E-64-d and MP may provide us with an effective strategy to take care of acute spinal cord injuries. However, further investigations into more underlying detailed mechanisms are still required, and the feasibility of the therapeutics for substantial neuron-protection against SCI in humans still needs to be elucidated.

## Abbreviations

AIF: Apoptosis inducing factor
bFGF: Basic fibroblast growth factor
EGPO: Endogenous peroxidase
GFAP: Glial fibrillary acidic protein
IL: Interleukin
MAP1B: Microtubule-associated protein 1B
MP: Methylprednisolone
NGF: Nerve growth factor
NFP: Nandrolone furylpropionate
SCI: Spinal cord injury
SD: Sprague Dawley
TdT: Terminal deoxynucleotidyl transferase
TUNEL: Terminal deoxynucleotidyl transferase-mediated deoxyuridine triphosphate-biotin nick end labeling.
